# Modern Homeopathy

**Published:** 1877-12

**Authors:** 


### EDITORIAL.

### MODERN HOMEOPATHY "DOES 'EM A GREAT DEAL OF GOOD."

A few weeks since we listened to an "exhaustive" dissertation upon Homeopathy by a member of our city Association, in which an effort was made to describe the principles of the Homeopathic practice of the present time. After it was finished the members seemed as ignorant and uncertain as to the real nature of Homeopathy as before. We thought we could describe and illustrate its principles and practice in two minutes, if the Society would permit relation of a bit of personal experience. Not long ago we were invited to visit a distinguished citizen of Buffalo, fatally ill at one of the most popular Homeopathic Water Cures of the State. We found in attendance a very gentlemanly, and we have no doubt well informed physician, of whom we would speak only in kindest and most respectful terms. After our consultation (if we may call it such,) we confidentially inquired of him: Now Doctor what do you do for all this company of invalids? He says, "Doctor, I had as soon tell you as not, we do not have many patients suffering from any real disease. For these ladies here, we order baths. Hot cold and medicated, and if we can get most of them to undress and dress themselves four or five times daily, they will soon be better, and the baths certainly do no harm. For the real sick ones we give all the medicines which you give, all that any physician gives and in the same doses. We give mercury, iodine, quinine, opium, and all other medicines deemed valuable by physicians, then we give our homeopathic remedies to boot. We take out one of these, (passing his fingers over his case of small bottles,) it makes no difference which one we select, and put a little of it into a tumbler, add some water and give them a teaspoon, telling them to take a teaspoonful every half hour." With evident satisfaction he says: "Now Doctor, *you* think homeopathy is a humbug, but *I* tell you it does 'em a great deal of good."

